# Quality and Specific Concerns of Clinical Guidelines for Integrated Chinese and Western Medicine: A Critical Appraisal

**DOI:** 10.1155/2020/9254503

**Published:** 2020-09-26

**Authors:** Xu Zhou, Sheng Xu, Qing Ren, Jianrong Chen

**Affiliations:** ^1^Evidence-Based Medicine Research Center, Jiangxi University of Traditional Chinese Medicine, Nanchang, Jiangxi, China; ^2^Department of Orthopedics and Traumatology, The University of Hong Kong, Pokfulam, Hong Kong, China; ^3^Department of Endocrinology, The First Hospital of Nanchang University, Nanchang, Jiangxi, China

## Abstract

**Objective:**

This study aimed to investigate the methodological quality of clinical guidelines (CGs) for integrated Chinese and Western medicine (ICWM) to inform clinical practice and guideline development.

**Methods:**

We searched PubMed, EMBASE, Chinese Biomedical Literature Database, China National Knowledge Infrastructure, Wanfang Data, VIP, five guideline databases, and four online book malls to identify ICWM CGs published up to January 11, 2019. Four independent appraisers assessed the quality of CGs using the Appraisal of Guidelines for Research and Evaluation II (AGREE II) instrument and evaluated six specific concerns for ICWM. The standardized scores were calculated for the individual AGREE II domains.

**Results:**

Sixty-two ICWM CGs were included. The median standardized scores in the six domains of AGREE II were 65% in scope and purpose, 46% in clarity of presentation, 26% in applicability, 24% in stakeholder involvement, 15% in rigor of development, and 0% in editorial independence. The quality of ICWM CGs was significantly associated with the publication year (higher quality for CGs published after 2014) and the development method (higher quality for evidence-based CGs). Only one ICWM CG obtained a direct recommendation for use, and 14 could be recommended for use after modifications. The intra-appraiser consistency of the AGREE II appraisal was good (mean intraclass correlation coefficient range, 0.813–0.998). ICWM CGs also lacked a systematic search of ancient traditional Chinese medicine (TCM) classics (40.3%), conversion of TCM recommendations from ancient Chinese to the vernacular (14.5%), a discussion of interactions between TCM and Western medicine (27.4%), and rankings of different ICWM choices (0%).

**Conclusions:**

Although an improvement after 2014 occurred, the current 64 ICWM CGs are generally of poor methodological quality. Only 15 ICWM CGs can be recommended for use directly or with modifications. As the key distinctions from Western/Chinese medicine CGs, the ICWM-specific recommendations are also insufficient for the ICWM CGs, especially for interactions between TCM and Western medicine and rankings of different ICWM choices. *Study Registration*. This study has been registered at PROSPERO (no. CRD42018095767).

## 1. Introduction

Although Western medicine has become the mainstay of the health care system in China, traditional Chinese medicine (TCM), an ancient medical approach with 3000 years of history, is still widely practiced [1, 2]. Integrated Chinese and Western medicine (ICWM) has proven to be more effective than either treatment style alone for many conditions, especially some chronic refractory conditions such as osteoarthritis and functional gastrointestinal disorders, as well as side effects from chemotherapy [3, 4]. In China, more than 90% of Western medicine practitioners and almost all TCM practitioners are simultaneously prescribing Chinese and Western medicine [5]. Moreover, ICWM has received specific support from the Chinese government as a basic health development strategy [6].

ICWM practice, however, is difficult. TCM is fundamentally different from Western medicine in both theory and practice. Western medicine is based on modern scientific methods and evidence, whereas TCM uses philosophical perspectives to explain physiological and pathological changes in the human body, as manifested in its unique diagnostic method of syndrome differentiation and use of Chinese herbs and acupuncture to treat diseases [7]. Due to the limitation of professional education, few healthcare practitioners master both Western medicine and TCM, and this is associated with ineffective application of ICWM and may increase costs and safety concerns [8]. In fact, most Western medicine practitioners do not understand TCM theory, such as syndrome differentiation, and the properties, compatibility, and contraindications of Chinese herbs [5]. Indeed, 21% of adverse events caused by TCM were associated with inappropriate combinations of Chinese and Western medicine [9]. TCM practitioners also lack evidence-based concepts and knowledge to make clinical decisions in Western medicine practice. They may be afraid that Western medicine is not sufficiently effective or safe and thus abandon this treatment approach, which also creates a risk of delaying treatment and missing the optimal time to treat a condition.

An essential approach to improving ICWM practice is to develop and utilize clinical guidelines (CGs), which are recommendation documents systematically developed to standardize clinical decisions in specific clinical settings [10]. To date, dozens of ICWM CGs have been released and have influenced decision-making and patient outcomes. The actual efficacy of ICWM CGs is, of course, closely related to their quality.

Many efforts have been made by methodologists to improve the quality of CGs. The Appraisal of Guidelines for Research and Evaluation II (AGREE II) instrument, developed in 2009, is a representative achievement of these efforts to assess the quality of CGs [11]. Owing to its advantages in structure and feasibility, AGREE II has a myriad of uses worldwide [12]. Overall, AGREE II is applicable to ICWM CGs, but many issues regarding the particular characteristics of ICWM, such as the vernacularization of ancient Chinese in TCM recommendations and the interactions between Chinese and Western medicine, should be further considered [13].

A previous study explored the quality of ICWM CGs published before 2014 [14]. However, this assessment was out of date and did not consider ICWM-specific concerns. With the publication of an increasing number of ICWM CGs since that previous study, we aimed to conduct an updated survey to inform clinical practice and guideline development by thoroughly investigating the methodological quality and ICWM-specific concerns of ICWM CGs.

## 2. Methods

### 2.1. Study Design

This is a survey of currently available ICWM CGs published before January 11, 2019, using the AGREE II instrument. The study protocol is registered at PROSPERO (no. CRD42018095767). We report this study following the Preferred Reporting Items for Systematic reviews and Meta-Analyses (PRISMA) criteria where applicable [15].

### 2.2. Searches

We searched PubMed, EMBASE, Chinese Biomedical Literature Database, China National Knowledge Infrastructure, Wanfang Data, VIP, five guideline databases (the National Guideline Clearinghouse, the Guidelines International Network, the National Institute for Health and Clinical Excellence, the Scottish Intercollegiate Guidelines Network, and MedLive), and four online book malls (http://www.amazon.com, http://www.jd.com, http://www.amazon.cn, and http://www.dangdang.com). The keywords used for the search included “integrated Chinese and Western,” “guideline,” “consensus,” and “recommendation,” among others (see details in Table S1 in the supplementary file).

### 2.3. Inclusion Criteria

We defined ICWM CGs as CGs that simultaneously focused on TCM and Western medicine for diagnosis or treatment. We used the World Health Organization's definition of CGs, i.e., systematic recommendations developed to assist any healthcare provider in making optimal clinical decisions [10]. The CGs were further divided into two types: (1) evidence-based CGs developed according to systematic searches and a summary of research evidence and (2) consensus-based guidelines developed according to scant research evidence or only expert opinions and consensus [16]. If a guideline had multiple versions, the final version was included. There were no restrictions regarding language.

### 2.4. Exclusion Criteria

The following CGs were excluded: (1) CGs developed for TCM or Western medicine alone; (2) no full text available; (3) translations; (4) CGs developed by a single author; (5) guideline-like textbooks; and (6) systematic reviews, narrative reviews, clinical pathways, and clinician training manuals.

### 2.5. Study Screening and Data Extraction

Two appraisers, independently and in duplicate, screened the bibliographies to identify potentially eligible CGs and then read the full text to determine the final eligibility. Discrepancies were resolved by discussion.

The following information was extracted from the CGs included using a standardized, pilot-tested form: authors, publication year, publication form, type of CG (evidence-based or consensus-based), type of developer, scope, condition, numbers of pages and references, consensus method, evidence grading system, composition of development team, and funding sources.

### 2.6. Appraisal of Guidelines

We used the AGREE II instrument to assess the quality of ICWM CGs across the following six domains: scope and purpose (3 items), stakeholder involvement (3 items), rigor of development (8 items), clarity of presentation (3 items), applicability (4 items), and editorial independence (2 items). The items in AGREE II are scored from strongly disagree (1 point) to strongly agree (7 points). A standardized score for each domain was calculated using the following formula: ((actual score − minimum score)/(maximum score − minimum score)) × 100%. Finally, we performed an overall assessment of “whether you recommend this guideline for use” with the following standards proposed by previous studies [17, 18]: (1) recommend, all domains scored ≥60%; (2) recommend with modifications, less than three domains scored <30% but one or more domains scored <60%; and (3) not recommend: three or more domains scored <30%.

Four appraisers who had experience in the development and appraisal of CGs independently assessed the quality of each CG. To enhance the consistency between the appraisers, they were trained in both English and Chinese versions of the AGREE II user's manual [19, 20], a paper of detailed explanations of the AGREE II assessment for the CGs of Chinese medicine [21], and an online AGREE II training tutorial (https://www.agreetrust.org/resource-centre/agree-ii/agree-ii-training-tools/). Before the formal appraisal, the appraisers performed a pilot exercise for two ICWM guidelines with different methodological qualities and discussed and addressed discrepancies. The formal appraisal was performed using an official online platform “My AGREE PLUS (https://http://www.agreetrust.org/my-agree/),” which is widely used in AGREE II appraisals and may enhance consistency between appraisers [22, 23].

To assess whether the CGs appropriately specified key concerns for ICWM, we consulted five clinicians each in Western medicine and Chinese medicine to collect the information they were most interested in and the gaps they most frequently faced when using ICWM guidelines. We determined the following six ICWM-specific concerns by a consensus meeting: (1) whether the CG included evidence from ancient TCM classics; (2) whether ancient Chinese in the TCM recommendations had been converted to the vernacular for the comprehension of non-TCM practitioners; (3) whether the CG provided the principles of the addition and subtraction of TCM interventions based on syndrome differentiation; (4) whether the CG specified the interactions between TCM and Western medicine; (5) whether the CG ranked the efficacy and safety of different ICWM interventions; and (6) whether the CG provided monitoring criteria for both diseases (Western medicine concept) and syndromes (TCM concept). All these items received an answer of “yes” or “no.” The assessment was also performed by four appraisers, and they achieved consistency through discussion.

### 2.7. Statistical Analysis

We calculated the mean, standard deviation, median, interquartile range (IQR), range, or proportion to describe the characteristics of the CGs, standardized AGREE II scores, and additional items. We performed stratified comparisons of CGs with different characteristics using the Mann–Whitney *U* test or the Kruskal–Wallis *H* test, and the difference was significant if the *p* value was less than 0.05. The intraclass correlation coefficient (ICC) and 95% confidence interval (CI) were calculated to assess consistency among the appraisers using a two-way mixed model; an ICC <0.50, 0.50–0.74, 0.75–0.89, and 0.90–1.00 indicated poor, fair, good, and excellent consistency, respectively [24].

## 3. Results

### 3.1. Epidemiological Characteristics

**Figure 1:**
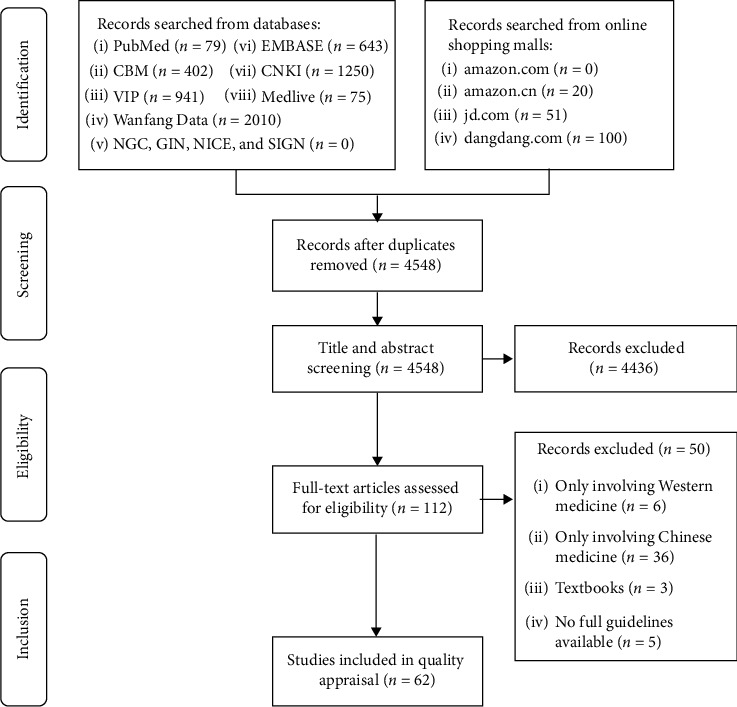
Flowchart of guideline screening. CBM = Chinese Biomedical Literature Database; CNKI = China National Knowledge Infrastructure; NGC = National Guideline Clearinghouse; GIN = Guidelines International Network; NICE = National Institute for Health and Clinical Excellence; SIGN = Scottish Intercollegiate Guidelines Network.

**Figure 2:**
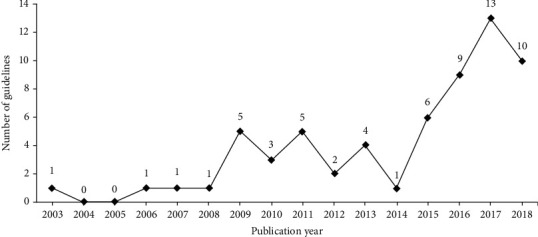
Annual trend of guideline publication.

As shown in Figure 1, the search yielded 5,571 results, and 62 ICWM CGs [25–86] were included in the quality appraisal after screening. The number of ICWM CGs was low between 2003 and 2015 but increased rapidly since 2016 (Figure 2). Most CGs were published as journal articles, except for one published as a conference paper. Two CGs were published in English, and the others were published in Chinese. There were 15 evidence-based and 47 consensus-based CGs. Most (87.1%) of the developers were academic associations, two (3.2%) were government departments, and six (9.7%) were nonofficial organizations. Recommendations for diagnosis and treatment were included in 47 CGs (75.8%), for only treatment in 13 (21.0%), and for only diagnosis in two (3.2%). Seventeen CGs (27.4%) focused on digestive diseases, 12 (19.4%) on cardiovascular diseases, 8 (12.9%) on skin diseases, 8 (12.9%) on urogenital diseases, 7 (11.3%) on respiratory diseases, and 10 (16.1%) on other diseases (e.g., bone, kidney, and nerve). The average number of pages and references in the included CGs was 6.2 and 32.3, respectively. Only five CGs were longer than 10 pages, and 13 had no references. The detailed characteristics of the included ICWM CGs are presented in Table S2 in the supplementary file.

### 3.2. AGREE II Appraisal

**Figure 3:**
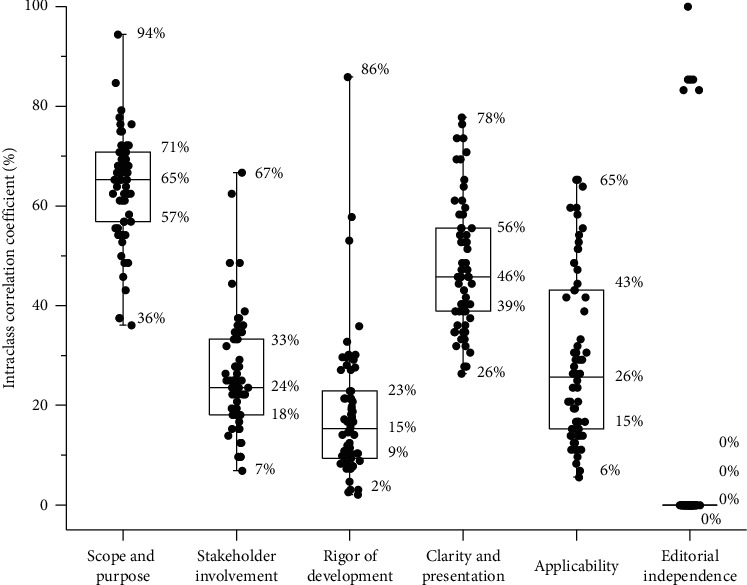
Standardized scores of AGREE II domains. Notes: the medial, bottom, and top of the boxes indicate medians, 25 percentiles, and 75 percentiles, and the bottom and top of the lines attached on the boxes indicate the minimums and maximums. Box plot cannot be generated for domain 6 because most guidelines scored 0. The dots indicate the individual score of each guideline.

The standardized AGREE II scores of the included CGs are shown in Figure 3 and Table S3 in the supplementary file.

#### 3.2.1. Scope and Purpose

This domain requires the CGs to explicitly describe the objectives and target population. The median score was 65.3% (IQR: 55.6–70.8), with 54 CGs (87.1%) scoring >50% but only two (3.2%) scoring >80%.

#### 3.2.2. Stakeholder Involvement

This domain requires the CGs to assemble a multidisciplinary guideline development team, consider the values and preferences of patients, and define target users. The median score was 23.6% (IQR: 18.1–33.3). Only two CGs (3.2%) scored >50%. Twenty-nine CGs (46.8%) provided author affiliations or specialties, but only 11 (17.7%) involved methodologists. No guidelines considered the values and preferences of patients.

#### 3.2.3. Rigor of Development

With regard to this domain, CGs should clearly describe the search strategy and rigorously assess the evidence. The median score was 14.6% (IQR: 9.4–21.4). Only three CGs scored >50%. Of the six CGs (9.7%) that reported the evidence grading system, two (3.2%) used the Grading of Recommendations Assessment, Development and Evaluation (GRADE) approach, and the others used less rigorous tools. To build consensus, 11 CGs (17.7%) used the Delphi method, and 5 (8.1%) used the meeting method.

#### 3.2.4. Clarity and Presentation

This domain requires the CGs to clearly present recommendations and provide multiple options for health issues. The median score was 45.8% (IQR: 37.5–55.6), and 23 CGs (37.1%) scored >50%. Thirteen CGs (21.0%) used tables or graphs to present the key recommendations.

#### 3.2.5. Applicability

For this domain, the CGs must consider facilitators or barriers of application and provide monitoring criteria. The median score was 25.0% (IQR: 13.9–43.1), and 11 CGs (17.7%) scored >50%. Only one CG provided a diagnostic manual as an additional tool to facilitate application.

#### 3.2.6. Editorial Independence

This domain requires the CGs to be free from competing interests. We were unable to calculate the median and IQR for this domain because most guidelines failed to report the relevant information. Six CGs (9.7%) reported nonprofit government funding or declared no competing interests and scored from 83.3% to 100%.

#### 3.2.7. Overall Assessment

Only one CG obtained a direct recommendation for use based on the criterion of a score greater than 60% in all six domains [27, 33, 73]. Fourteen CGs [27–30, 33, 38, 45, 50, 54, 71, 79, 81, 85, 86] could be recommended for use after improving the quality of one or two domains. The other 48 CGs were not directly recommended for use because of obvious flaws in more than three domains.

#### 3.2.8. Stratified Comparison

Table 1 presents the results of stratified analyses. The CGs published after 2014 had significantly higher scores in five domains (stakeholder involvement, rigor of development, clarity and presentation, applicability, and editorial independence) than those published in 2014 or before. Evidence-based CGs were significantly better than consensus-based CGs in all domains except applicability. There were no significant differences in quality between CGs published in different forms (journals indexed in the Chinese Science Citation Database versus others) and between those published by different developers (official organizations vs. nonofficial organizations).

#### 3.2.9. Consistency

Overall, the intra-appraiser consistency was good in all AGREE II domains, as indicated by ICC values ranging from 0.813 to 0.998 (Table 2).

### 3.3. ICWM-Specific Concerns

Based on the assessment of ICWM-specific concerns, 25 (40.3%) guidelines cited ancient TCM classics, but only 9 (14.5%) adequately converted ancient Chinese to plain text in the TCM recommendations. Of the 60 treatment CGs, 31 (50.0%) provided the principles on addition and subtraction of TCM interventions, 17 (27.4%) described interactions between TCM and Western medicine, and 14 (22.6%) provided monitoring criteria for both diseases and TCM syndromes. No guideline included recommendations on ranking the efficacy and safety of different integrated treatment choices. The detailed results are presented in Table S4 in the supplementary file.

## 4. Discussion

This study is a significant update of a previous study on the quality of ICWM CGs. We used a precise definition of ICWM CGs and performed a comprehensive search; as a result, we added 32 new and 9 updated ICWM CGs to those detailed in the previous survey. With good intra-appraiser consistency, our results revealed that the overall methodological quality of ICWM CGs is poor. Only one ICWM CG was directly recommended for use, and fourteen CGs can be recommended after modifications. Compared with 626 Western medicine CGs [87], the ICWM CGs had obviously lower scores across four AGREE II domains (ICWM CGs vs. Western medicine CGs: stakeholder involvement, 24% vs. 35%; rigor of development, 15% vs. 43%; clarity and presentation, 46% vs. 60%; and editorial independence, 0% vs. 30%) and similar scores in the other two domains (scope and purpose, 65% vs. 64%; applicability, 22% vs. 24%). Stratified analysis showed better quality among ICWM CGs published after 2014 and those developed with evidence-based methods.

The rigor of development is a vital component ensuring the quality of CGs, but the score for this domain was very low across the ICWM CGs. Using an evidence-based framework to develop CGs allows for the systematic identification of the current evidence, a rigorous and transparent assessment, and the development of evidence-informed recommendations, enhancing the quality of CGs [88]. The stratified analysis also indicated overall better quality among evidence-based CGs than among consensus-based CGs. The use of a reasonable evidence grading system is crucial for identifying high-quality evidence [89], and since its initiation, the GRADE approach has been regarded as the preferred tool for comprehensively and rigorously grading the quality of evidence [90]. We found that the GRADE approach was used in only two ICWM CGs, both of which obtained the highest scores and a direct recommendation for use, demonstrating a significant role of GRADE in improving the quality of CGs.

Using the publication year of a previous study assessing the quality of ICWM CGs as a time node, we found that CG quality in five domains was better for those published after 2014 than those published during 2014 or before. This subgroup difference should also be associated with the better quality of evidence-based CGs; in fact, most (93.3%) evidence-based CGs included in the study were published after 2014. We consider that there were two possible reasons for the increasing tendency of publishing evidence-based CGs. First, the amount of high-grade evidence for ICWM such as systematic reviews increased rapidly after 2014, which is the foundation for developing an evidence-based CG. Second, the methodology of evidence-based CG development (e.g., the AGREE II and the GRADE tools) became more complete and popular in China after 2014.

Both evidence- and consensus-based CGs require consensus methods to formulate recommendations. The formal consensus methods include the Delphi method, the nominal group method, and consensus-building meetings [91]. Some ICWM CGs reported the consensus methods used (the Delphi method or consensus-building meetings) but provided no details on the number of rounds, construction of the team of experts, and standards to reach consensus. More ICWM CGs claimed that the recommendations were formulated based on multiple expert discussions, without providing a specific method. This is also a main reason for the low scores in the rigor of development domain.

There were particularly conspicuously low scores in the editorial independence domain. In general, the development of a CG requires considerable physical resources and financial support, which may be associated with potential bias (e.g., a drug whose developer provided funding may be preferentially recommended). Nevertheless, because there are generally no requirements for a conflicts of interest statement for publication [92], most ICWM CGs published in Chinese journals had no such important statements.

This survey also investigated six ICWM-specific concerns that represent key differences between ICWM guidelines and guidelines for Western or Chinese medicine alone and are associated with a better methodological quality of ICWM CGs. As a traditional medical science, TCM practice is highly dependent on evidence from ancient classics [9]. Despite the fact that 40.3% of ICWM CGs involved ancient TCM classics, no guidelines described the search strategy, eligibility criteria, or methods of quality assessment; thus, it can be speculated that the ancient TCM classics were not compiled in a systematic manner. Moreover, text in ancient Chinese is common in TCM recommendations [93], but only 16.1% of CGs adequately converted such text to vernacular text. In China, most Western medicine practitioners cannot easily understand ancient Chinese. Therefore, a translation of ancient Chinese may facilitate ICWM clinical practice.

A growing controversy in TCM is the potential toxicity of Chinese herbs [13]. Hence, potential interaction effects of ICWM, especially those with safety concerns, should be specified in ICWM CGs. Nonetheless, a majority of ICWM CGs presented Western medicine and TCM recommendations separately or included only a synoptic description of the general principles of ICWM treatment without specific details on potential interactions. From the clinicians' perspective, we would hope to find content in guidelines regarding a rank of efficacy and safety of different ICWM choices for specific clinical circumstances. Indeed, relevant evidence, such as comparative cost-effectiveness research and network meta-analyses, is scant in the ICWM field. In general, guideline developers should strive to produce this evidence rather than to abandon such recommendations.

There are some limitations of this study. Although we searched electronic databases and online book malls, some ICWM CGs that have not been published but are being used in clinical practice may have been omitted. We empirically consider these CGs to be of low quality. Moreover, because AGREE II does not provide detailed criteria, we used a nonofficial standard that has been applied in several previous studies for the overall assessment (i.e., whether a CG can be recommended for use), but the judgment boundary in the standard is not fully recognized.

Although the quality of ICWM CGs has been improving, there are still substantial methodological gaps. We have several suggestions for future ICWM CGs based on the results of this study. First, assembling an interdisciplinary team, including Western medicine experts, TCM experts, economists, patient representatives, and methodologists familiar with guideline development methodology and AGREE II, would help to reduce methodological limitations. Additionally, a methodology training program for all team members before development may be helpful. Second, we strongly recommend using the GRADE approach to assess the quality of evidence and formulate recommendations. A specialized evidence grading system is warranted for assessing ancient TCM classics. Third, all ICWM CGs need to expand upon recommendations regarding ICWM-specific concerns, especially regarding interactions between Western medicine and TCM and a ranking of recommendations based on comprehensive considerations of efficacy and safety. Finally, obligatory requirements for disclosing the role of funders and conflicts of interest are indispensable for improving the editorial independence of ICWM CGs.

## 5. Conclusion

The current 62 ICWM CGs are generally of poor methodological quality, especially in terms of stakeholder involvement, rigor of development, applicability, and editorial independence. The quality of CGs published after 2014 and evidence-based CGs are relatively high. Only one and fourteen ICWM CGs could be recommended for use directly and with modifications, respectively. Most ICWM CGs fail to provide important ICWM-specific concerns, including ancient TCM classic evidence, translation of ancient Chinese, interactions between TCM and Western medicine, and rankings of different ICWM choices. Rigorous methodology and ICWM-specific considerations are thus warranted in future ICWM CGs to improve their quality and applicability.

## Figures and Tables

**Table 1:** Comparisons of different subgroups on AGREE II domain scores.

Subgroups	Scope and purpose	Stakeholder involvement	Rigor of development	Clarity and presentation	Applicability	Editorial independence
Overall	65.3 (55.6, 70.8)	23.6 (18.1, 33.3)	14.6 (9.4, 21.4)	45.8 (37.5, 55.6)	25.0 (13.9, 43.1)	0 (0, 0)

Publication year						
2014 or before (*n* = 24)	61.8 (54.2, 69.1)	18.8 (14.3, 25.0)	9.7 (7.3, 12.4)	40.3 (33.7, 47.9)	20.2 (13.9, 29.2)	0 (0, 0)
After 2014 (*n* = 38)	66.7 (60.0, 70.8)	27.1 (23.3, 35.1)^b^	21.1 (14.1, 29.3)^b^	49.3 (40.3, 61.1)^b^	30.6 (16.7, 51.4)^a^	0 (0, 100)^a^

Development method						
Evidence-based (*n* = 15)	68.1 (65.3, 70.8)	34.7 (22.2, 44.4)	27.1 (21.4, 35.9)	58.3 (45.8, 70.8)	30.6 (16.7, 51.4)	0 (0, 100)
Consensus-based (*n* = 47)	62.5 (54.2, 70.8)^a^	23.6 (18.1, 26.4)^b^	11.5 (8.3, 18.2)^b^	41.7 (36.1, 52.8)^b^	23.6 (13.9, 43.1)	0 (0, 85.0)^b^

Publication form						
CSCD-indexed journals (*n* = 36)	66 (61.5, 72.2)	25 (18.4, 34.7)	16.7 (9.9, 21.3)	45.8 (37.9, 58.3)	20.8 (14.3, 31.6)	0 (0, 100)
Others (*n* = 26)	63.2 (54.2, 69.8)	23.6 (18.1, 27.8)	12.8 (8.1, 27.7)	45.8 (39.3, 54.6)	29.2 (15.0, 53.2)	0 (0, 85.0)

Developer						
Official organizations (*n* = 56)	65.3 (56.9, 70.5)	24.3 (19.8, 33.0)	15.4 (9.4, 22.5)	45.8 (38.9, 55.6)	26.4 (15.7, 43.1)	0 (0, 85.0)
Nonofficial organizations (*n* = 6)	63.2 (37.5, 80.6)	18.8 (15.7, 43.8)	13.8 (10.0, 38.7)	37.5 (27.5, 74.7)	14.6 (13.9, 30.9)	0 (0, 100)

Note: CSCD = Chinese Science Citation Database. Superscripts a and b indicate that the *p* values of the Mann–Whitney *U* test are <0.05 and <0.01, respectively. Data are presented as median and interquartile range for the former five domains and as median and range for the editorial independence domain.

**Table 2:** Intraclass correlation coefficient in the AGREE II appraisal.

Domains	Intraclass correlation coefficient (95% confidence interval)
Scope and purpose	0.813 (0.765–0.853)
Stakeholder involvement	0.915 (0.894–0.934)
Rigor of development	0.914 (0.900–0.925)
Clarity and presentation	0.903 (0.879–0.924)
Applicability	0.882 (0.856–0.904)
Editorial independence	0.998 (0.998–0.999)

## Data Availability

The data used to support the findings of this study are available from the corresponding author upon request.

## Abbreviations

AGREE II: Appraisal of Guidelines for Research and Evaluation II
CI: Confidence interval
CG: Clinical guideline
GRADE: Grading of Recommendations Assessment, Development and Evaluation
ICC: Intraclass correlation coefficients
ICWM: Integrated Chinese and Western medicine
IQR: Interquartile range
PRISMA: Preferred Reporting Items for Systematic Reviews and Meta-Analyses
TCM: Traditional Chinese medicine.

## Supplementary Materials

Supplementary Materials Table S1: search strategy. Table S2: characteristics of integrated Chinese and Western medicine guidelines. Table S3: assessment results of AGREE II. Table S4: assessment results of IWCM-specific concerns.
