# Supplementary material for: Quality and Specific Concerns of Clinical Guidelines for Integrated Chinese and Western Medicine: A Critical Appraisal
Source: Evid Based Complement Alternat Med. 2020 Sep 26;2020:9254503. doi: 10.1155/2020/9254503 (PMC7533756; doi:10.1155/2020/9254503)
Supplement: Supplementary Materials — Table S1: search strategy. Table S2: characteristics of integrated Chinese and Western medicine guidelines. Table S3: assessment results of AGREE II. Table S4: assessment results of IWCM-specific concerns. [file 9254503.f1.pdf]

Table S1. Search strategy

Search strategy in English databases	Search strategy in Chinese databases
1. Medicine, Chinese Traditional [MeSH]	1. 医学，中国传统[主题词] # medicine, traditional Chinese [MeSH]
2. integrated traditional Chinese [all fields]	2. 中西医结合[主题词] # integrated Chinese and Western medicine [MeSH]
3. integrative traditional Chinese [all fields]	3. 中西医结合疗法[主题词] # integrated Chinese and Western treatment [MeSH]
4. integrated Chinese [all fields]	4. 中医[所有字段] # traditional Chinese medicine [all fields]
5. integrative Chinese [all fields]	5. 中西医[所有字段] # integrated Chinese and Western medicine [all fields]
6. combination of traditional Chinese and Western [all fields]	6. 1 OR 2 OR 3 OR 4 OR 5
7. combination of Chinese and Western [all fields]	7. 指南[主题词] # guideline [MeSH]
8. combination of Chinese traditional and Western [all fields]	8. 规范[主题词] # standard [MeSH]
9. 1 OR 2 OR 3 OR 4 OR 5 OR 6 OR 7 OR 8	9. 手册[主题词] # manual [MeSH]
10. Guidelines as Topic [MeSH]	10. 指南[所有字段] # guideline [all fields]
11. guideline [publication type]	11. 规范[所有字段] # standard [all fields]
12. Standard of Care [MeSH]	12. 手册[所有字段] # manual [all fields]
13. Consensus [MeSH]	13. 共识[所有字段] # consensus [all fields]
14. Manuals as Topic [MeSH]	14. 推荐[所有字段] # recommendation [all fields]
15. manual [MeSH]	15. 7 OR 8 OR 9 OR 10 OR 11 OR 12 OR 13 OR 14
16. guideline* [all fields]	16. 6 AND 15
17. manual* [all fields]	
18. consensus* [all fields]	
19. handbook* [all fields]	
20. recommendation [all fields]	
21. 10 OR 11 OR 12 OR 13 OR 14 OR 15 OR 16 OR 17 OR 18 OR 19 OR 20	
22. 9 AND 21	

Note: Explanations after "#" are the English translation. The medical subject heading terms are only available in the PubMed, EMBASE, and Chinese Bio-medical Literature Database.

Table S2. Characteristics of Integrated Chinese and Western Medicine Guidelines

Topic of guideline	Year	Scope	No. of references	No. of pages	Form of publication	Developer	Development method	Involvement of Methodologist	Consensus-building method	Evidence grading system
Acne [25]	2015	T	0	6	CSCD journal	Non-official organization	CB	NR	NR	No
Acute fever in children [26]	2012	T	14	4	Non-CSCD journal	Non-official organization	CB	No	NR	No
Acute myocardial infarcation [27]	2018	D & T	163	13	CSCD journal	CMDA	EB	Yes	NR	GRADE
Acute pancreatitis [28]	2017	D & T	50	9	Non-CSCD journal	CAITCWM	CB	Yes	3 rounds Delphi	No
Atherosclerosis [29]	2017	D & T	85	5	Non-CSCD journal	CAITCWM	EB	No	Delphi	No
Benign prostatic hyperplasia [30]	2017	D & T	39	6	CSCD journal	CAITCWM	EB	No	NR	Highly-credible literatures were selected, without specific criteria
Cardiac rehabilitation after coronary artery bypass grafting [31]	2017	T	33	4	CSCD journal	CNCC	CB	Yes	NR	No
Cardiopulmonary resuscitation [32]	2007	T	14	6	Non-CSCD journal	JSSTCM	CB	NR	NR	No
Cerebral infarction [33]	2018	D & T	134	9	CSCD journal	CAITCWM	EB	Yes	NR	Revised Jadad scale*
Children cough [34]	2010	D & T	10	5	CSCD journal	Non-official organization	CB	NR	NR	No
Children Mycoplasma pneumonia [35]	2017	D & T	38	5	CSCD journal	CACM	CB	No	NR	No
Cholelithiasis [36]	2018	D & T	35	7	Non-CSCD journal	CAITCWM	CB	NR	Delphi	No

(Continued Table S2)

Topic of guideline	Year	Scope	No. of references	No. of pages	Form of publication	Developer	Development method	Involvement of Methodologist	Consensus-building method	Evidence grading system
Chronic atrophic gastritis [37]	2018	D & T	92	11	Non-CSCD journal	CAITCWM	CB	NR	3 rounds Delphi	No
Chronic cardiac failure [38]	2016	D & T	50	9	CSCD journal	CAITCWM	EB	Yes	NR	Clinical evidence was based on systematical review, without specific criteria
Chronic gastritis [39]	2012	D & T	30	6	CSCD journal	CAITCWM	CB	NR	Meeting	No
Chronic non-atrophic gastritis [40]	2018	D & T	34	8	Non-CSCD journal	CAITCWM	CB	NR	NR	No
Chronic prostatitis [41]	2015	D & T	66	9	CSCD journal	CAITCWM	EB	No	NR	Highly-credible literatures were selected, without specific criteria
Chronic renal failure [42]	2015	D & T	7	11	CSCD journal	CAITCWM	CB	NR	NR	No
Community acquired pneumonia [43]	2016	T	48	6	Non-CSCD journal	SHSIM	CB	No	NR	No
Corticosteroid-dependent dermatitis [44]	2009	D & T	8	2	CSCD journal	CMDA	CB	NR	NR	No
Dyslipidemia [45]	2017	D & T	0	7	Non-CSCD journal	CAITCWM	EB	NR	NR	Evidence selection was based on the principal of evidence-based medicine, without specific criteria
Eczema [46]	2015	T	17	3	CSCD journal	CAITCWM	CB	No	NR	No
Eczema and dermatitis [47]	2009	D & T	0	1	Non-CSCD journal	WASCM	CB	NR	NR	No

(Continued Table S2)

Topic of guideline	Year	Scope	No. of references	No. of pages	Form of publication	Developer	Development method	Involvement of Methodologist	Consensus-building method	Evidence grading system
Erectile dysfunction [48]	2016	D & T	67	7	CSCD journal	CAITCWM	EB	No	NR	Oxford Evidence grading method*
Functional constipation [49]	2018	D & T	102	9	Non-CSCD journal	CAITCWM	CB	Yes	Delphi	No
Functional dyspepsia [50]	2017	D & T	31	6	Non-CSCD journal	CAITCWM	CB	NR	Delphi	No
Gastroesophageal reflux disease [51]	2011	D & T	16	4	CSCD journal	CAITCWM	CB	NR	Meeting	No
Haemorrhoids [52]	2006	D & T	0	3	CSCD journal	CMA	CB	NR	NR	No
Hand-foot-and-mouth disease [53]	2010	D & T	0	3	Non-CSCD journal	MHPRC	CB	NR	NR	No
Hepatic fibrosis [54]	2017	D & T	28	6	Non-CSCD journal	CAITCWM	EB	NR	3 rounds Delphi	Evidence grading methods of American Society of Hepatology*
Herpes progenitalis [55]	2009	D & T	0	2	Non-CSCD journal	CAITCWM	CB	NR	NR	No
Human avian influenza [56]	2016	D & T	37	7	CSCD journal	CAITCWM	CB	NR	NR	No
Hypertensive cerebral hemorrhage [57]	2016	D & T	0	8	Non-CSCD journal	BJAIM	EB	Yes	NR	No
IgA nephropathy [58]	2013	D	11	7	Conference paper	CAITCWM	CB	NR	Delphi	No
Influenza A virus subtype H1N1 [59]	2010	D & T	0	5	Non-CSCD journal	MHPRC	CB	NR	NR	No
Irritable bowel syndrome [60]	2011	D & T	13	4	CSCD journal	CAITCWM	CB	NR	Meeting	No
Leucoderma [61]	2009	T	0	2	CSCD journal	CAITCWM	CB	NR	NR	No
Liver cirrhosis [62]	2011	D & T	6	3	Non-CSCD journal	CAITCWM	CB	NR	NR	No
Liver cirrhosis with ascites [63]	2011	D & T	9	4	CSCD journal	CAITCWM	CB	NR	Meeting	No

(Continued Table S2)

Topic of guideline	Year	Scope	No. of references	No. of pages	Form of publication	Developer	Development method	Involvement of Methodologist	Consensus-building method	Evidence grading system
Male infertility [64]	2015	D & T	19	5	CSCD journal	CAITCWM	EB	No	NR	High-quality evidence was selected, without specific criteria
Melasma [65]	2016	T	21	4	CSCD journal	CAITCWM	CB	No	NR	No
Multiple organ dysfunction syndrome in elderly [66]	2014	D & T	26	5	CSCD journal	CAITCWM	CB	No	NR	No
Myelodysplastic syndrome [67]	2013	T	15	3	CSCD journal	Non-official organization	CB	No	NR	No
Non-alcoholic fatty liver disease [68]	2017	D & T	27	7	Non-CSCD journal	CAITCWM	CB	NR	NR	No
Osteoarthritis [69]	2013	D & T	0	6	CSCD journal	CAITCWM	CB	NR	NR	No
Petic ulcer [70]	2018	D & T	48	9	Non-CSCD journal	CAITCWM	CB	NR	3 rounds Delphi	No
Polycystic ovary syndrome [71]	2017	D	107	8	CSCD journal	Non-official organization	CB	No	Meeting	No
Premature ejaculation [72]	2018	D & T	38	6	CSCD journal	CAITCWM	CB	No	NR	No
Primary liver cancer [73]	2018	D & T	105	13	CSCD journal	Non-official organization	EB	Yes	Delphi	GRADE
Psoriasis vulgaris [74]	2009	D & T	0	1	Non-CSCD journal	WASCM	CB	NR	NR	No
Psycho-cardiological disease [75]	2017	D & T	0	4	Non-CSCD journal	CAITCWM	CB	NR	NR	No
Recurrent vulvovaginal candidiasis [76]	2017	T	24	4	CSCD journal	CAITCWM	EB	NR	NR	No
Rosacea [77]	2016	T	25	4	CSCD journal	CAITCWM	CB	No	NR	No

(Continued Table S2)

Topic of guideline	Year	Scope	No. of references	No. of pages	Form of publication	Developer	Development method	Involvement of Methodologist	Consensus-building method	Evidence grading system
Sepsis [78]	2013	D & T	9	8	CSCD journal	CAITCWM	CB	No	NR	No
Severe acute pancreatitis [79]	2015	D & T	18	5	Non-CSCD journal	CAITCWM	EB	NR	NR	Criteria of the International Sepsis Forum*
Severe acute respiratory syndrome [80]	2003	D & T	64	22	CSCD journal	CMA	EB	NR	NR	No
Stage 1 Cardiac rehabilitation [81]	2017	T	17	9	CSCD journal	CNCC	CB	Yes	NR	No
Syndrome of blood statis [82]	2011	D & T	41	6	CSCD journal	CAITCWM	CB	Yes	Delphi	No
Ulcerative colitis [83]	2018	D & T	48	8	Non-CSCD journal	CAITCWM	CB	NR	NR	No
Urolithiasis [84]	2008	D & T	0	4	Non-CSCD journal	CAITCWM	CB	No	NR	No
Xinmailong injection for chronic cardiac failure [85]	2016	T	46	5	CSCD journal	CAITCWM	CB	Yes	NR	No
Zika virus disease [86]	2016	D & T	19	4	CSCD journal	CAITCWM	CB	NR	NR	No

Abbreviations: BJAIM=Beijing Association of Integrative Medicine; CACM=China Association of Chinese Medicine; ; CAITCWM=Chinese Association of the Integration of Traditional Chinese and Western Medicine; CB=consensus-based; CMA=Chinese Medical Association; CMDA=Chinese Medical Doctor Association; CNCC=Chinese National Cardiovascular Center; CSCD=Chinese Science Citation Database; D=diagnosis; EB=evidence-based; GRADE=The Grading of Recommendations Assessment, Development and Evaluation system; JSSTCM=Jiang-Su Society of Traditional Chinese Medicine; MHPRC=Ministry of Health of the People's Republic of China; NR=not reported; SHSIM=Shanghai Society of Integrated Medicine; T=treatment; WASCM=the Whole Army Society of Chinese Medicine

*: The grade of evidence was only associated with type of studies in the grading system.

Table S3. Assessment results of AGREE II

Topic of guideline	Standardized scores of AGREE II (%)						Overall assessment
	D1	D2	D3	D4	D5	D6	
Acne [25]	76.4	18.1	17.2	77.8	19.4	0.0	C
Acute fever in children [26]	37.5	19.4	10.4	26.4	13.9	0.0	C
Acute myocardial infarcation [27]	94.4	62.5	53.1	70.8	20.8	83.3	B
Acute pancreatitis [28]	68.1	23.6	32.8	40.3	58.3	0.0	B
Atherosclerosis [29]	70.8	36.1	35.9	33.3	13.9	85.4	B
Benign prostatic hyperplasia [30]	66.7	34.7	15.6	47.2	41.7	0.0	B
Cardiac rehabilitation after coronary artery bypass grafting [31]	70.8	34.7	17.2	51.4	11.1	0.0	C
Cardiopulmonary resuscitation [32]	69.4	25.0	7.3	54.2	13.9	0.0	C
Cerebral infarction [33]	68.1	34.7	57.8	63.9	30.6	85.4	B
Children cough [34]	50.0	16.7	10.4	31.9	15.3	0.0	C
Children Mycoplasma pneumonia [35]	72.2	22.2	14.1	45.8	20.8	0.0	C
Cholelithiasis [36]	54.2	26.4	28.1	54.2	59.7	0.0	C
Chronic atrophic gastritis [37]	72.2	25.0	27.6	55.6	63.9	0.0	C
Chronic cardiac failure [38]	69.4	48.6	29.7	69.4	30.6	0.0	B
Chronic gastritis [39]	56.9	25.0	16.7	55.6	47.2	0.0	C
Chronic non-atrophic gastritis [40]	48.6	23.6	8.3	47.2	52.8	0.0	C
Chronic prostatitis [41]	68.1	22.2	18.8	58.3	51.4	0.0	C
Chronic renal failure [42]	75.0	31.9	9.9	37.5	27.8	0.0	C
Community acquired pneumonia [43]	55.6	37.5	14.1	45.8	8.3	0.0	C
Corticosteroid-dependent dermatitis [44]	62.5	9.7	7.3	38.9	11.1	0.0	C
Dyslipidemia [45]	68.1	44.4	27.1	76.4	41.7	83.3	B
Eczema [46]	65.3	19.4	15.1	30.6	26.4	0.0	C
Eczema and dermatitis [47]	54.2	18.1	3.1	36.1	26.4	0.0	C
Erectile dysfunction [48]	62.5	22.2	21.4	45.8	31.9	0.0	C
Functional constipation [49]	55.6	23.6	22.9	40.3	59.7	0.0	C
Functional dyspepsia [50]	70.8	29.2	30.2	61.1	48.6	0.0	B
Gastroesophageal reflux disease [51]	61.1	25.0	12.5	38.9	25.0	0.0	C
Haemorrhoids [52]	62.5	15.3	9.9	34.7	5.6	0.0	C
Hand-foot-and-mouth disease [53]	54.2	15.3	2.6	45.8	6.9	0.0	C
Hepatic fibrosis [54]	61.1	27.8	30.2	44.4	51.4	0.0	B
Herpes progenitalis [55]	70.8	23.6	10.4	45.8	23.6	0.0	C
Human avian influenza [56]	43.1	18.1	10.4	59.7	12.5	0.0	C
Hypertensive cerebral hemorrhage [57]	65.3	18.1	21.4	52.8	19.4	0.0	C
IgA nephropathy [58]	69.4	22.2	14.6	27.8	11.1	0.0	C
Influenza A virus subtype H1N1 [59]	66.7	15.3	3.1	52.8	33.3	0.0	C
Irritable bowel syndrome [60]	56.9	22.2	17.7	40.3	23.6	0.0	C
Leucoderma [61]	63.9	13.9	7.8	45.8	15.3	0.0	C
Liver cirrhosis [62]	54.2	25.0	10.9	33.3	29.2	0.0	C
Liver cirrhosis with ascites [63]	68.1	25.0	12.0	40.3	38.9	0.0	C
Male infertility [64]	66.7	33.3	9.9	34.7	12.5	0.0	C

(Continued Table S3)

Topic of guideline	Standardized scores of AGREE II (%)						Overall assessment
	D1	D2	D3	D4	D5	D6	
Melasma [65]	62.5	22.2	19.3	41.7	16.7	0.0	C
Multiple organ dysfunction syndrome in elderly [66]	76.4	20.8	8.3	48.6	16.7	0.0	C
Myelodysplastic syndrome [67]	37.5	12.5	8.9	27.8	13.9	0.0	C
Non-alcoholic fatty liver disease [68]	36.1	23.6	8.9	34.7	44.4	0.0	C
Osteoarthritis [69]	61.1	9.7	7.3	48.6	29.2	0.0	C
Petic ulcer [70]	65.3	27.8	21.4	65.3	54.2	0.0	C
Polycystic ovary syndrome [71]	79.2	36.1	22.9	43.1	13.9	85.4	B
Premature ejaculation [72]	65.3	25.0	18.2	38.9	20.8	0.0	C
Primary liver cancer [73]	84.7	66.7	85.9	73.6	65.3	100	A
Psoriasis vulgaris [74]	48.6	6.9	2.1	40.3	26.4	0.0	C
Psycho-cardiological disease [75]	70.8	27.8	11.5	40.3	15.3	0.0	C
Recurrent vulvovaginal candidiasis [76]	56.9	26.4	27.1	58.3	16.7	0.0	C
Rosacea [77]	45.8	23.6	16.7	52.8	11.1	0.0	C
Sepsis [78]	72.2	33.3	9.4	36.1	16.7	0.0	C
Severe acute pancreatitis [79]	70.8	12.5	29.2	55.6	16.7	0.0	C
Severe acute respiratory syndrome [80]	77.8	37.5	21.4	73.6	65.3	0.0	B
Stage 1 Cardiac rehabilitation [81]	75.0	38.9	20.8	61.1	43.1	0.0	B
Syndrome of blood statis [82]	63.9	18.1	19.8	31.9	9.7	0.0	C
Ulcerative colitis [83]	52.8	23.6	9.4	69.4	55.6	0.0	C
Urolithiasis [84]	58.3	9.7	4.7	44.4	29.2	0.0	C
Xinmailong injection for chronic cardiac failure [85]	66.7	48.6	29.7	38.9	43.1	0.0	B
Zika virus disease [86]	62.5	33.3	7.8	34.7	30.6	0.0	B

Note: D1: Scope and purpose; D2: Stakeholder involvement; D3: Rigor of development; D4: Clarity and presentation; D5: Applicability; D6: Editorial independence. Results of overall assessment: A: recommended for use; B: recommend for use after modification; C: Not recommended for use.

Table S4. Assessment results of IWCM-specific concerns

Topic of guideline	Q1	Q2	Q3	Q4	Q5	Q6
Acne [25]	No	No	Yes	No	No	No
Acute fever in children [26]	Yes	No	Yes	Yes	No	No
Acute myocardial infarcation [27]	Yes	No	Yes	No	No	No
Acute pancreatitis [28]	Yes	No	Yes	Yes	No	Yes
Atherosclerosis [29]	No	No	Yes	No	No	No
Benign prostatic hyperplasia [30]	Yes	No	No	No	No	No
Cardiac rehabilitation after coronary artery bypass grafting [31]	No	No	No	No	No	No
Cardiopulmonary resuscitation [32]	No	No	Yes	No	No	No
Cerebral infarction [33]	No	No	Yes	No	No	No
Children cough [34]	Yes	Yes	No	Yes	No	No
Children Mycoplasma pneumonia [35]	No	Yes	No	No	No	No
Cholelithiasis [36]	Yes	No	Yes	Yes	No	Yes
Chronic atrophic gastritis [37]	No	No	Yes	Yes	No	Yes
Chronic cardiac failure [38]	No	No	Yes	No	No	No
Chronic gastritis [39]	No	No	Yes	Yes	No	Yes
Chronic non-atrophic gastritis [40]	Yes	No	Yes	Yes	No	Yes
Chronic prostatitis [41]	Yes	Yes	No	Yes	No	Yes
Chronic renal failure [42]	No	No	No	No	No	No
Community acquired pneumonia [43]	No	No	Yes	No	No	No
Corticosteroid-dependent dermatitis [44]	Yes	No	Yes	No	No	No
Dyslipidemia [45]	No	No	No	No	No	Yes
Eczema [46]	No	No	Yes	No	No	No
Eczema and dermatitis [47]	No	No	No	No	No	No
Erectile dysfunction [48]	Yes	No	No	Yes	No	No
Functional constipation [49]	Yes	No	Yes	Yes	No	Yes
Functional dyspepsia [50]	Yes	No	Yes	Yes	No	Yes
Gastroesophageal reflux disease [51]	No	No	No	No	No	No
Haemorrhoids [52]	No	Yes	No	No	No	No
Hand-foot-and-mouth disease [53]	No	No	No	No	No	No
Hepatic fibrosis [54]	Yes	No	No	No	No	No
Herpes progenitalis [55]	No	No	Yes	No	No	No
Human avian influenza [56]	No	No	Yes	No	No	No
Hypertensive cerebral hemorrhage [57]	Yes	Yes	Yes	Yes	No	No
IgA nephropathy [58]	No	No	N/A	N/A	N/A	N/A
Influenza A virus subtype H1N1 [59]	No	No	Yes	No	No	No
Irritable bowel syndrome [60]	No	No	Yes	No	No	No
Leucoderma [61]	No	No	No	No	No	No
Liver cirrhosis [62]	Yes	No	Yes	No	No	Yes
Liver cirrhosis with ascites [63]	No	No	Yes	No	No	Yes
Male infertility [64]	Yes	No	No	No	No	No

(Continued Table S4)

Topic of guideline	Q1	Q2	Q3	Q4	Q5	Q6
Melasma [65]	No	No	No	No	No	No
Multiple organ dysfunction syndrome in elderly [66]	No	No	No	No	No	No
Myelodysplastic syndrome [67]	No	No	Yes	No	No	No
Non-alcoholic fatty liver disease [68]	Yes	No	Yes	No	No	Yes
Osteoarthritis [69]	No	No	No	Yes	No	No
Petic ulcer [70]	Yes	No	Yes	Yes	No	Yes
Polycystic ovary syndrome [71]	No	No	N/A	N/A	N/A	N/A
Premature ejaculation [72]	Yes	No	No	No	No	No
Primary liver cancer [73]	No	No	No	Yes	No	No
Psoriasis vulgaris [74]	No	No	No	No	No	No
Psycho-cardiological disease [75]	Yes	No	Yes	No	No	No
Recurrent vulvovaginal candidiasis [76]	Yes	No	No	No	No	No
Rosacea [77]	Yes	No	No	No	No	No
Sepsis [78]	No	No	No	Yes	No	No
Severe acute pancreatitis [79]	No	No	No	No	No	No
Severe acute respiratory syndrome [80]	Yes	Yes	Yes	No	No	No
Stage 1 Cardiac rehabilitation [81]	No	Yes	No	No	No	No
Syndrome of blood statis [82]	No	No	No	No	No	No
Ulcerative colitis [83]	Yes	No	Yes	Yes	No	Yes
Urolithiasis [84]	No	Yes	No	No	No	No
Xinmailong injection for chronic cardiac failure [85]	Yes	Yes	No	No	No	No
Zika virus disease [86]	No	No	Yes	No	No	No

Q1: Whether the CPG included evidence from ancient TCM classics?

Q2: Whether the ancient Chinese in the TCM recommendations had been converted to the vernacular for the comprehension of non-TCM practitioners?

Q3: Whether the CPG provided the principles of the addition and subtraction of TCM interventions based on syndrome differentiation?

Q4: Whether the CPG specified the interactions between TCM and Western medicine?

Q5: Whether the CPG ranked the efficacy and safety of different ICWM interventions?

Q6: Whether the CPG provided monitoring criteria for both diseases (Western medicine concept) and syndromes (TCM concept)?

Abbreviations: ICWM = integrated Chinese and Western medicine; TCM = traditional Chinese medicine; N/A = Not applicable
